# Telerehabilitation for Neurological Motor Impairment: A Systematic Review and Meta-Analysis on Quality of Life, Satisfaction, and Acceptance in Stroke, Multiple Sclerosis, and Parkinson’s Disease

**DOI:** 10.3390/jcm13010299

**Published:** 2024-01-04

**Authors:** Sara Federico, Luisa Cacciante, Błażej Cieślik, Andrea Turolla, Michela Agostini, Pawel Kiper, Alessandro Picelli

**Affiliations:** 1 Laboratory of Healthcare Innovation Technology, IRCCS San Camillo Hospital, 30126 Venice, Italy; sara.federico@hsancamillo.it (S.F.); luisa.cacciante@hsancamillo.it (L.C.); blazej.cieslik@hsancamillo.it (B.C.); 2 Department of Biomedical and Neuromotor Sciences—DIBINEM, Alma Mater Studiorum Università di Bologna, Via Massarenti, 9, 40138 Bologna, Italy; andrea.turolla@unibo.it; 3 Unit of Occupational Medicine, IRCCS Azienda Ospedaliero—Universitaria di Bologna, 40138 Bologna, Italy; 4 Rehabilitation Unit, Department of Neuroscience, University—General Hospital of Padova, 35128 Padova, Italy; michela.agostini@unipd.it; 5 Department of Neurosciences, Biomedicine and Movement Sciences, University of Verona, 37100 Verona, Italy; alessandro.picelli@univr.it; 6 Canadian Advances in Neuro-Orthopedics for Spasticity Congress (CANOSC), Kingston, ON K7K 1Z6, Canada

**Keywords:** stroke, multiple sclerosis, Parkinson’s disease, telerehabilitation, QoL, satisfaction, technology acceptance

## Abstract

Telerehabilitation (TR) seems to be a viable and feasible solution to face the rehabilitative challenges posed by neurological impairments and to improve patients’ quality of life (QoL). This review aims to synthesize and analyze the evidence on the impact of physiotherapy intervention through TR on QoL in patients with stroke, Parkinson’s disease (PD), and multiple sclerosis (MS), together with an evaluation of their satisfaction and technology acceptance levels. Through a systematic search of the literature and a screening process, treatment effects were assessed with meta-analyses using the standardized mean difference, setting the confidence interval at 95%. We included 28 studies in the review, which were analyzed for methodological quality, whereas 16 studies were included in the meta-analyses. The results suggest a significant improvement in QoL in patients who underwent TR. We were unable to perform analyses for satisfaction and technology acceptance outcomes due to insufficient data. Overall, motor TR has a positive impact on the QoL of patients with neurological diseases, especially in stroke patients; although caution is needed in the interpretation of the results due to the high heterogeneity found. For PD and MS, TR seems to yield comparable results to in-person treatment.

## 1. Introduction

### 1.1. Description of the Conditions

Neurological motor impairments resulting from stroke, multiple sclerosis (MS), and Parkinson’s disease (PD) pose significant challenges to affected individuals and to healthcare systems. Stroke, a leading cause of adult disability globally, is often associated with partial or complete paralysis on one side of the body [1]. MS, characterized by the demyelination of nerve fibers, results in a wide range of motor impairments, including muscle weakness, spasticity, and ataxia [2,3]. PD, primarily known for its motor symptoms such as tremors, rigidity, and bradykinesia, significantly impacts an individual’s ability to perform everyday tasks [4]. All these conditions have in common the long-term consequences the disease brings with it, resulting in chronic impairments that require long-term multidisciplinary management. However, healthcare systems are still struggling to answer to the rehabilitative needs of people with neurological impairments. In addition to this, neurological diseases have a major impact not only on the different functions of affected individuals (e.g., motor, speech, language, cognitive impairments), but also on their quality of life (QoL) [3,5,6]. Indeed, while it is known that there is an issue for healthcare systems in providing a certain continuity of care for individuals with neurological conditions at an adequate dose [7], the perceived QoL levels of individuals with stroke, PD, and MS seem to decrease drastically [3,5,6].

### 1.2. Description of the Intervention

Telerehabilitation (TR), a dynamic and evolving branch of telemedicine, has emerged as a promising approach to provide comprehensive rehabilitation services remotely [8]. By using technological advancements, TR is uniquely positioned to address the different impairments associated with these neurological conditions, offering a wide range of therapeutic exercises, educational resources, and emotional support in patients’ own homes, by providing synchronous (i.e., online, with the presence of the therapist in real time) and asynchronous (i.e., by monitoring patients’ training) treatments [9]. The possibility for the patient to have treatment at home in a synchronous or asynchronous modality therefore has positive effects not only in terms of the dose of treatment that can be delivered and the possibility to continue the rehabilitation program at home, but also in terms of QoL, which in turn could have positive effects on functional improvements. Indeed, treatment conducted within patients’ social, educational, and vocational environments can lead to improved functional outcomes and enhanced family and community integration [10]. Emerging evidence suggests that TR may hold promise as an effective alternative to conventional treatment for various neurological disorders. TR has been shown to be as effective as conventional rehabilitation for motor, cortical, and mood disorders in stroke survivors [7,11,12,13]. Additionally, non-immersive virtual reality (VR)-based TR is a promising approach for improving static and dynamic balance and gait in people with PD and MS [14,15,16]. Likewise, TR may be beneficial for prolonging and maintaining the goals achieved during rehabilitation [12].

### 1.3. Why It Is Important to Conduct This Review

Despite the growing interest in TR, there remains a critical gap in the literature regarding its comprehensive impact on the QoL, satisfaction, and acceptance among individuals affected by stroke, MS, and PD. The focus on these aspects is crucial, given the multifaceted nature of these neurological conditions and the different impairments they encompass. Understanding how TR can improve QoL, increase patient satisfaction with the treatment process, and enhance the acceptance of these novel approaches is essential for ensuring comprehensive and effective care for individuals grappling with the complex challenges posed by these neurological conditions. This review contributes to the enhancement of knowledge regarding TR and its impact on QoL in neurological conditions. Its implications serve as a useful resource for researchers, clinicians, and healthcare providers, offering insights that contribute to the ongoing efforts to enhance the well-being of individuals dealing with complex neurological challenges.

### 1.4. Objectives

This systematic review and meta-analysis aim to synthesize and analyze existing evidence on the role of motor TR in improving QoL levels, together with the investigation of the satisfaction and acceptance levels in patients with neurological impairments (i.e., stroke, MS, PD) who underwent TR.

## 2. Materials and Methods

This systematic review with a meta-analysis was conducted according to the PRISMA guidelines [17], and the protocol was registered a priori in the PROSPERO database under the following registration number: CRD42021276763.

### 2.1. Electronic Searches

We conducted a comprehensive search for articles written in English in PubMed, Embase, Web of Science, and the Cochrane Library. We included studies without time restrictions, with the last search conducted on 24 October 2022. A detailed description of the search strategy is presented in Appendix A.

### 2.2. Study Selection

In this review, we planned to include (1) studies designed as randomized controlled trials (RCT), quasi-randomized controlled trials (quasi-RCTs), and controlled clinical studies (CCTs), with (2) adults (>18 years) diagnosed with stroke, PD, and MS, undergoing (3) physiotherapy interventions based on TR (e.g., home-based rehabilitation conducted via TR, including interventions delivered through computers, virtual reality, and video conferencing) as compared to (4) conventional therapies (e.g., exercises, mobilizations). Eventually, (5) the primary outcome of interest was the assessment of QoL. Secondary outcomes included levels of patient satisfaction and technology acceptance. We excluded studies with healthy individuals or with subjects affected by other neurological or neurodegenerative pathologies and those not involving physiotherapy TR interventions. Furthermore, studies not comparing TR intervention to conventional therapies and not assessing QoL, satisfaction levels, or acceptance of TR technology were excluded. For study selection through abstract screening after duplicates’ removal, two independent reviewers conducted the screening of the records, based on titles and abstracts, using the Rayyan tool [18]. A third reviewer was selected to solve any disagreements. At the end of this process, full texts of the records were obtained, and the same procedure was used for full text screening and for the assessment of the methodological quality of the studies (i.e., risk of bias assessment).

### 2.3. Outcomes

The primary outcome of this review was the QoL, as defined by the World Health Organization (WHO) as the multidimensional perception of an individual’s state of physical, mental, and social well-being within their personal, cultural context and in relation to the values upon which they base their goals, expectations, standards, and concerns [19]. This definition denotes an ideal state, with a concept that requires the construction of indicators capable of capturing the many subjective and functional dimensions of well-being. Outcome measures considered consisted of questionnaires related to QoL for the different pathologies included in the review (e.g., stroke impact scale [SIS] for stroke, Parkinson’s disease questionnaire-8 [PDQ-8] for PD, multiple sclerosis quality of life 54 [MSQOL-54] for MS). Satisfaction levels and technological acceptance of TR systems were evaluated as secondary outcomes, assessed with specific questionnaires (e.g., stroke-specific patient satisfaction with care [SSPSC], client satisfaction questionnaire [CSQ]).

### 2.4. Data Extraction and Management

A specific synoptic table was created and filled with data extracted from the included studies. The following study details were extracted:

1. Citation details: authors, year of publication;
2. Aim of the study;
3. Study type;
4. Participant details (e.g., diagnosis, age, gender distribution, disease severity, months/years since the event, number of patients per group);
5. Intervention (i.e., type and dose);
6. TR method (e.g., hardware, software, and type of connection, delivery mode);
7. Assessment time points;
8. Outcome measures (related to our study objectives: QoL, satisfaction, and acceptance);
9. Conclusions of the studies.

### 2.5. Assessment of Risk of Bias in Included Studies

The included articles were qualitatively analyzed by two independent reviewers using the Revised Cochrane Tool Risk of Bias 2 (RoB2) [20] and the Cochrane Tool Risk of Bias in Non-Randomized Studies of Interventions (ROBINS-I) [21]. A third reviewer solved any disagreements. Through RoB2, we assessed the following domains: (1) selection bias, encompassing sequence generation and allocation concealment; (2) detection bias, examining the blinding of outcome assessment; (3) attrition bias, addressing incomplete outcome data; and (4) reporting bias, focusing on selective reporting. Each domain’s risk of bias was coded as ‘high risk’ in the presence of a significant likelihood of bias, ‘low risk’ in cases with a low probability of bias, and ‘unclear risk’ when a precise determination of bias incidence was uncertain. For non-randomized studies, we used ROBINS-I, with which we evaluated the following coded biases: confounding bias, participant selection, intervention classification, deviations from intended interventions, missing data, measurement of outcomes, and selection of reported results. Each domain was judged with a “low risk”, “moderate risk”, “serious risk”, or “critical risk” of bias. The overall risk of bias for each study was then summarized by considering judgments across all domains.

### 2.6. Measures of Treatment Effect

We used Review Manager 5.4 (RevMan 2020) [22] to conduct the review and to perform statistical analyses. Given the varied measurement scales of outcomes, treatment effects were assessed using the standardized mean difference (SMD). The confidence interval (CI) for continuous outcomes was set at 95%. For satisfaction and technological acceptance outcomes, quantitative results could not be obtained due to the nature of their data; hence, they are described in a narrative manner.

### 2.7. Dealing with Missing Data

In the presence of missing data or data not reported as means and standard deviations, we contacted trial authors to ask for them (e.g., information and/or data reported as means and standard deviations to carry out the meta-analyses). Whenever feasible, we converted available data using the procedures outlined in Section 6.5.2.2 of the Cochrane Handbook for Systematic Reviews of Interventions [23]. If we did not receive a response and we were not able to extract this kind of data, the article was included in the review but excluded from the meta-analysis.

### 2.8. Subgroup Analysis and Investigation of Heterogeneity

We planned to perform subgroup analysis according to neurological diseases (i.e., stroke, PD, MS). Statistical heterogeneity was assessed with the *I^2^* statistic, establishing the cut-off value at 50%.

### 2.9. Data Synthesis

We conducted meta-analyses based on a random-effects model, based on the presence of heterogeneity, with 95% CI using RevMan 5.4. We explored heterogeneity as detailed above.

## 3. Results

### 3.1. Results of the Search

**Figure 1:**
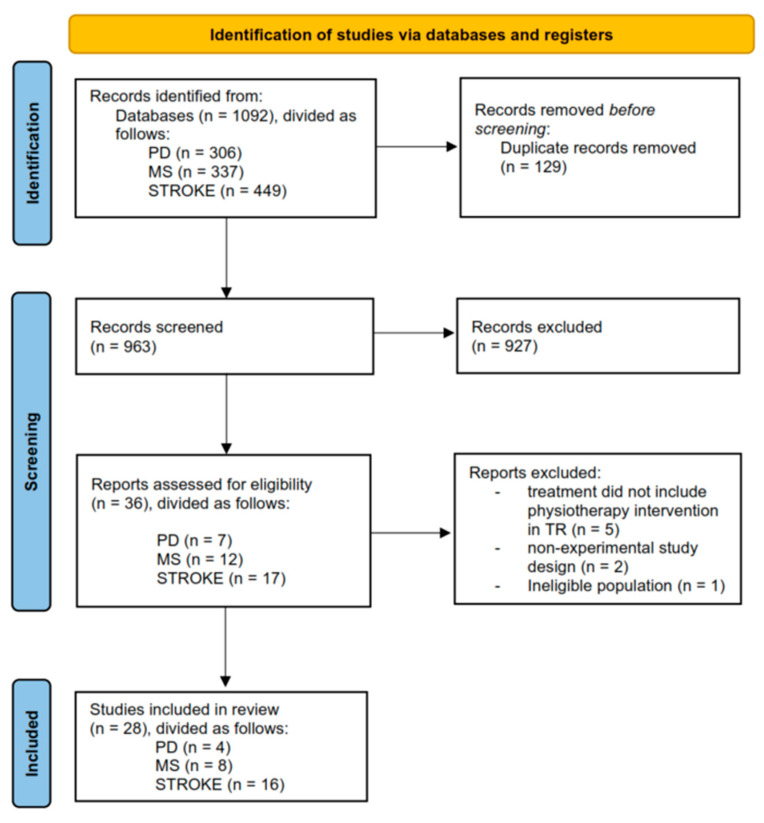
PRISMA flow diagram.

The database search yielded a total of 1092 results from four electronic databases. After removing duplicates, 963 abstracts were screened using the Rayyan tool. Subsequently, 36 studies were included for full-text screening. After full-text screening, 28 studies met the inclusion criteria for qualitative analysis. At the end of the process, 16 studies were included for quantitative analysis. The PRISMA flowchart of the review process is shown in Figure 1.

### 3.2. Included Studies

All three pathologies were represented in the selected studies, including stroke (n = 16) [24,25,26,27,28,29,30,31,32,33,34,35,36,37,38,39], MS (n = 8) [16,40,41,42,43,44,45,46], and PD (n = 4) [47,48,49,50,51]; among the 28 included studies, 26 were RCTs and 2 [25,50] were CCTs, investigating the efficacy of TR for addressing motor impairments.

The overall number of participants across all trials was 1659, with 884 individuals enrolled in TR programs, while 775 participants received conventional treatments. Among the TR modality used, asynchronous TR interventions were the most prevalent, appearing in 13 studies [16,24,26,28,33,36,41,43,44,47,48,50,51]; synchronous TR was used in 5 studies [32,34,39,42,49], whereas mixed TR approaches, thus combining both synchronous and asynchronous elements, were employed in 10 studies [25,27,29,30,31,35,37,40,45,46]. In all the studies, TR appeared to be safe, feasible, and a valid alternative to face-to-face intervention. In 11 studies with asynchronous TR delivery, participants reported satisfaction and QoL levels comparable to those obtained with conventional rehabilitation [16,24,33,36,41,43,44,47,48,50,51]. In the remaining 2 studies [25,28], TR led to superior results compared to conventional therapy with respect to the outcome of interest. The treatments used in asynchronous modality were heterogeneous: VR platforms [16,36], even combined with sensors for the detection of vital parameters [47], wireless motion sensors, and motion capture technology [24,50,51]. Web platforms [43,44], mobile apps [28,48], SMS or e-mail messaging systems [26], and robotic devices [33] were also used. Among the studies with mixed TR delivery, eight demonstrated results comparable to those obtained with conventional rehabilitation [25,27,30,31,37,40,45,46], and in the remaining two studies [29,35], TR turned out to be inferior to conventional rehabilitation, when assessing satisfaction and QoL levels. In this delivery type, the real-time TR interaction was via video-conferencing or phone call [45,46], combined with sensors [30,31,40], messaging devices [29,35], and in some cases with caregiver supervision [25]. In two studies, dedicated platforms were used [25,27]. In all the studies with synchronous delivery, TR was defined as being as effective as conventional rehabilitation regarding the outcomes of interest. In relation to this delivery modality, video-conferencing was used [32,39,42] in combination with VR-based exercises with a balance board [49] or motion-tracking system [34]. A detailed description of the included studies is presented in Table S1 in the Supplementary Materials.

### 3.3. Excluded Studies

After full-text screening, we excluded a total of eight studies. Two studies [52,53] were considered ineligible due to their non-experimental nature, whereas another five studies [54,55,56,57,58] were excluded as they did not involve any physiotherapy or motor treatments but solely focused on tele-visits. One study [59] was excluded because it included patients with stroke, as well as patients with severe acquired brain injuries and traumatic brain injuries, without providing a separate analysis of their results.

### 3.4. Risk of Bias in Included Studies

**Figure 2:**
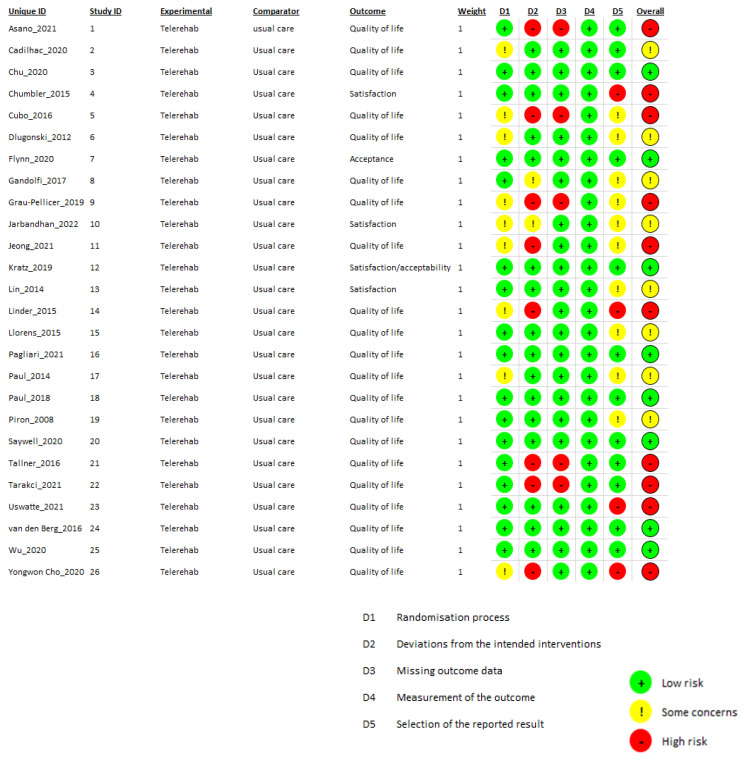
Risk of bias of the included studies (RCTs).

The 26 randomized clinical trials were analyzed using the RoB2 tool, and the synthesis of the results is graphically presented in Figure 2; the remaining 2 non-randomized trials were analyzed using the ROBINS-I tool, and the detailed description of the evaluation is presented in Table 1.

#### 3.4.1. Risk of Bias in Randomized Studies

- Bias arising from the randomization process: 17 studies [16,24,28,29,32,34,35,36,37,39,42,43,44,45,46,48,49,51] received a low risk of bias while the other 9 raised some concerns. These concerns primarily stemmed from the lack of information regarding participant allocation blinding during the randomization phase. In addition, in one study [26], the experimental group was statistically more active at the baseline; in three studies [27,41,47], some information regarding how the randomization process was conducted was missing. Baseline data for the participants were missing in one study [27] and in two [33,40] the randomization process was adjusted to balance the two groups or to follow the personal preferences of the participants.
- Bias due to deviations from intended interventions: In 16 studies, the domain received a low risk-of-bias rating. Two studies [31,49] raised some concerns among reviewers, and eight studies received a high risk-of-bias rating due to the exclusion of some data from the final analysis for a high number of patients who did not complete the study [24,27,30,33,41,45,46,47].
- Bias due to missing outcome data: Twenty-one studies received a low risk-of-bias rating. Five studies received a high risk-of-bias rating [24,30,45,46,47]. The reason for this rating was the same as for the previous domain.
- Bias in measurement of the outcome: All studies in this domain received a low risk-of-bias judgement.
- Bias in selection of the reported result: Twelve studies received a low risk-of-bias judgment. Ten studies raised some concerns, and four studies received a high risk-of-bias judgment. Seven studies modified data from the protocol, introducing variations in assessment scales, outcomes, and the expected timepoints for evaluations. These modifications led to a high risk-of-bias judgment in four studies [28,29,33,36], and raised concerns in two studies [30,31].

#### 3.4.2. Risk of Bias in Non-Randomized Studies

The study performed by Benvenuti et al. [25] was judged with a critical risk of bias due to the pooling of the two groups and subsequent data combination after trial completion. Additionally, there was a substantial 26.5% overall data loss, and no statistical method was used to analyze the data of participants who did not complete the study. The study conducted by Isernia et al. [50] was judged to have a serious risk of bias due to the unbalanced nature of the two groups from the outset (3:1), and the restriction of the first assessment analysis to the experimental group only.

### 3.5. Effect of Interventions

#### 3.5.1. Comparison 1. TR versus Conventional Treatment in Stroke, MS, and PD—Outcome: QoL

**Figure 3:**
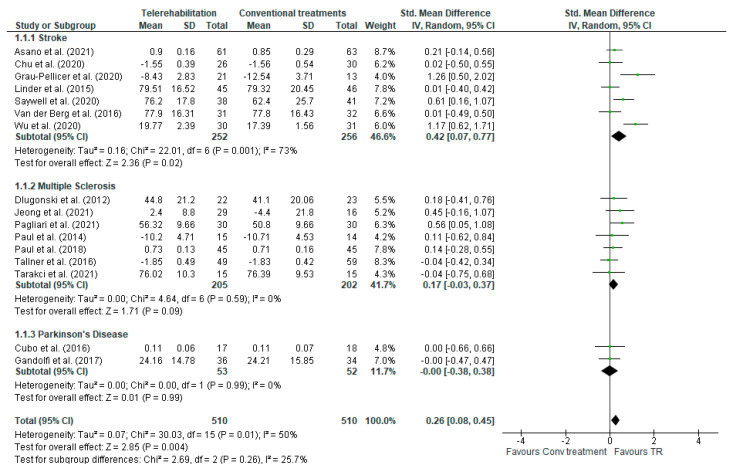
Comparison 1. TR vs. conventional treatment in stroke, MS, and PD. Outcome: QoL.

A total of sixteen studies, with an overall number of 1208 participants, were analysed, to evaluate the improvement in QoL levels. To account for the heterogeneity arising from distinct pathologies within the dataset, subgroup analysis was conducted for each pathology of interest (i.e., stroke, MS, and PD). The analyses were performed using the standardized mean difference (SMD) with a random effect model, since all the included studies used different outcome measures for the same outcome. A statistically significant difference was found in favour of the stroke subgroup [SMD (95% C.I) = 0.41 (0.12, −0.70), *I*^2^ = 68%] and in total comparison [SMD (95% C.I) = 0.28 [0.11, −0.44], *I*^2^ = 48%]. No significant difference was found in either the MS [SMD (95% C.I) = −0.17 (0.03, −0.37), *I*^2^ = 0%] or PD [SMD (95% C.I) = −0.00 (−0.38, 0.38), *I*^2^ = 0%] subgroups (Figure 3).

#### 3.5.2. Comparison 2. Synchronous TR versus Conventional Treatment in Stroke and PD—Outcome: QoL

**Figure 4:**
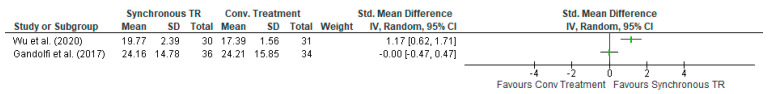
Comparison 2. Synchronous TR vs. conventional treatment. Outcome: QoL.

To assess the effects of synchronous TR on QoL compared to conventional treatment, two studies were included in the analysis, one for stroke [SMD (95% C.I) = 1.17 (0.62, 1.71)] and one for PD [SMD (95% C.I) = −0.00 (−0.47, −0.47)]. The results of individual studies were reported as they could not be combined due to pathology heterogeneity (Figure 4).

#### 3.5.3. Comparison 3. Asynchronous TR versus Conventional Treatment in Stroke and MS—Outcome: QoL

**Figure 5:**
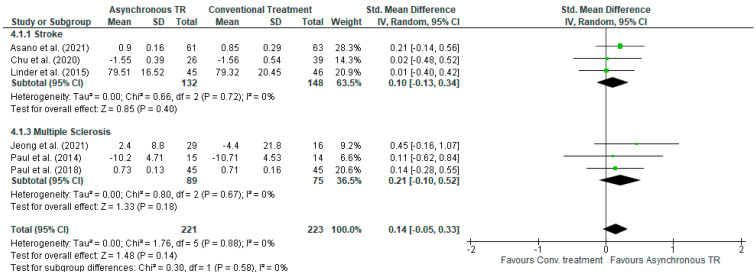
Comparison 3. Asynchronous TR vs. conventional treatment in stroke and MS. Outcome: QoL.

Seven studies with an overall number of 444 subjects were included in the meta-analysis for the effect of asynchronous telerehabilitation on QoL compared to conventional treatments. No significant differences were found between the two groups, in either the overall effect [SMD (95% C.I) = 0.14 (−0.05, −0.33), *I^2^* = 0%] or in the subgroups of stroke effect [SMD (95% C.I) = 0.10 (−0.13, 0.34), *I^2^* = 0%] or MS [SMD (95% C.I) = 0.21 (−0.10, 0.52), *I^2^* = 0%] (Figure 5).

#### 3.5.4. Comparison 4. Mixed TR versus Conventional Treatment-Subgroups Stroke and MS—Outcome: QoL

**Figure 6:**
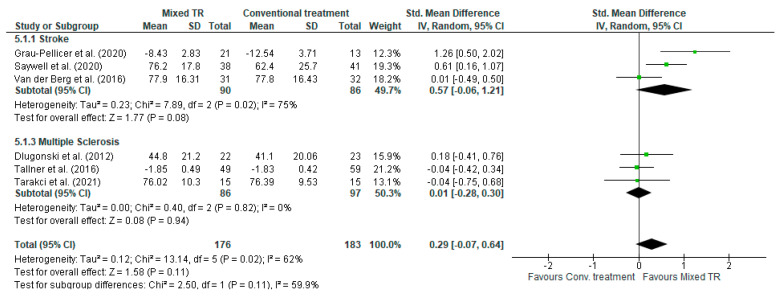
Comparison 4. Mixed TR vs. conventional treatment in stroke and MS. Outcome: QoL.

A total of six studies with 359 participants were included in the comparison between mixed TR and conventional treatments for the improvement of QoL. No statistically significant differences were found for either the overall effect [SMD (95% C.I) = 0.29 (−0.07, 0.64), *I*^2^ = 61%] or the stroke [SMD (95% C.I) = 0.57 (−0.06, −1.21), *I*^2^ = 75%] or MS [SMD (95% C.I) = 0.01 (−0.28, −0.30) *I*^2^ = 0%] subgroups (Figure 6).

### 3.6. Narrative Synthesis

Acceptance and satisfaction levels of the technology were primarily assessed through a variety of measurements, predominantly qualitative in nature, rendering quantitative analyses unfeasible. TR emerged as a viable and well-accepted approach, demonstrating a satisfaction level comparable to that of conventional treatments. These outcomes were analysed through various measurements, mostly qualitative, and therefore, quantitative analyses were not feasible. Table A1 (Appendix B) provides the summary of the overall 17 included studies that measured these outcomes along with their respective results.

#### 3.6.1. Effects of Telerehabilitation Compared to Conventional Treatment for Improving Patients’ Satisfaction

In the narrative synthesis of studies investigating satisfaction associated with TR use, a total of 13 studies have been included. To assess satisfaction, mainly ad hoc questionnaires [27,30,31,34,40,43,47,49] or personalized and modified versions of existing models [29] were used, as well as official assessment scales [38]. Additionally, structured interviews [25] and surveys [26,36] were conducted.

#### 3.6.2. Effects of Telerehabilitation Compared to Conventional Treatment for Acceptance

A total of eight studies measured technological acceptance. For the assessment of acceptance, official questionnaires [25,42] and semi-structured telephone interviews [44] were employed. Additionally, it was evaluated through ad hoc questionnaire, and, in one study, it was inferred from the actual number of treatment hours [24].

## 4. Discussion

With this review with meta-analyses, we aimed to synthesise and analyse the current evidence on the impact of motor TR on QoL levels in neurological diseses (i.e., stroke, MS, and PD), together with an evaluation of acceptance levels and satisfaction with the technology.

Our meta-analysis indicates that motor TR has a significant and overall positive effect on QoL in patients with neurological diseases with a moderate level of heterogeneity (*I*^2^ = 48%) across studies. Within the stroke subgroup, the overall effectiveness of TR is notably significant, pointing to a substantial impact on improving QoL. However, the high heterogeneity (*I*^2^ = 68%) suggests that there is a considerable variability among the studies in this subgroup. This variability may be attributed to differences in interventions, treatment dosage, assessment time points, methodological quality of the study, and study size. Therefore, the high heterogeneity in the stroke subgroup warrants careful interpretation of the overall treatment effect.

Within the PD and MS subgroups, no significant differences were observed, demonstrating that TR yields comparable effects to traditional treatment in improving the QoL for patients with these neurodegenerative pathologies. However, despite the absence of heterogeneity, the limited number of studies in these two subgroups makes it difficult to draw firm conclusions on the comparability of these two modalities for QoL improvement. In the comparisons between synchronous TR and traditional treatment, asynchronous TR, and traditional treatment, as well as mixed TR and traditional treatment, we did not find differences in the improvement of QoL. Examining the overall effect, there seems to be a slightly potentially greater effect in mixed TR as compared to in-person treatment. Furthermore, a subtle positive trend toward asynchronous TR was noted in MS, as well as a positive trend for mixed TR in stroke patients. However, these trends should be approached with caution and further investigation is warranted, particularly in specific comparisons between different treatment methodologies, to better understand their effects on the QoL.

Despite the controversial findings between studies in which some authors found a beneficial effect of TR [25,28], in contrast with other researchers who reported that TR led to inferior results as compared to conventional treatment for QoL and satisfaction levels [29,35], it seems to be a general consesus in considering TR as non inferior to conventional, in-person treatments. This finding is consistent with the present literature on the topic that is focusing on evaluating the effect of TR on clinical outcomes [7,60,61]. Nevertheless, as TR is a modality with which we are delivering treatments, attention has to be paid not only to clinical outcomes, but also to the effect that this kind of modality can bring into patients’ lives. Indeed, if we think about TR as a modality of treatment delivery, the direct consequence is to think about the content of the exercises and the training proposed through it, which should be evidence-based and with a clinical effect that has already been documented. With this regard, Laver and colleagues already pointed out that “in theory, the mechanisms leading to recovery should mirror those associated with conventional rehabilitation programmes” [62]. Given these premises, it is important to look at the strength that TR can bring with it. One of these is, indeed, the benefit to patients’ QoL, given the fact that they are at home, in their vocational environment, with their family and carers, and that they do not need to travel to reach the rehabilitation centres. Thus far, studies that have assessed this aspect of TR are limited and with small sample sizes. However, when pooling together data from single studies on the topic, we found that TR positively impacted the QoL levels of patients with neurological impairments, especially in stroke patients. Our study reveals a heightened attention given to stroke within the current body of research, followed by MS and PD. This heightened attention could stem from various factors such as the prevalence of stroke, its impact on patients, or the potential efficacy of TR interventions in addressing the unique challenges posed by stroke-related impairments. Additionally, our analysis indicates a prevalent utilization of asynchronous interventions as the primary mode of TR delivery. This preference may be associated with the adaptability and convenience offered by asynchronous approaches, allowing for tailored interventions, flexibility, and resource optimization. Furthermore, the type of treatment administered, the outcome measures, the technology used, the study size, the treatment intensity, and the duration of follow-ups exhibit heterogeneity even within the same treatment population subgroup. This variability highlights the complexity of implementing TR interventions; recognizing and addressing this heterogeneity is pivotal for advancing our understanding of TR effectiveness and optimizing its application across different clinical scenarios.

When interpreting the results of our meta-analyses, it is imperative to consider the methodological quality of the included studies. As we can observe, the overall methodological quality of the studies raises some concerns when trying to interpret our results and to give some recommendations. The reliability of our findings hinges on the methodological robustness of the individual studies, emphasizing the need for cautious interpretation of our results. Furthermore, it is essential to recognize the differences among MS, stroke, and PD, as these conditions have distinct characteristics that may influence the study’s outcomes. Acknowledging the chronic nature of MS and PD, in contrast to the acute nature of stroke, and considering variations in treatments and their diverse impacts on QoL are crucial. Addressing these distinctions as potential limitations ensures a more accurate and transparent interpretation of the study’s findings. Additionally, some QoL measures may lack appropriate validation in the telehealth setting and may not exhibit good correlation with each other [5]. It is important to acknowledge that the use of patient-reported outcomes as a measure of disability is a limitation, as there may be significant divergence compared to physician-assessed outcomes [63].

Further studies investigating aspects related to the QoL of neurologic patients who undergo physiotherapy treatments in TR are needed, in order to foster the implementation of TR as a modality with which we could guarantee a certain continuity of care and accessibility to rehabilitation services, together with a beneficial effect on patients’ QoL, which could in turn have a positive impact on their functional performance.

### Study Limitations

The limitations of the present study include a notable heterogeneity across studies, a disproportionate representation of stroke, MS, and PD studies, and the presence of a limited number of studies demonstrating a high methodological quality, which may impact the reliability of the conclusions drawn from this analysis.

## 5. Conclusions

Our study demonstrated that motor TR shows a positive and significant impact on the QoL for patients with neurological diseases, including stroke, PD, and MS. The effectiveness is particularly notable in stroke patients, although caution is needed in the interpretation of this result due to the high heterogeneity found in this subgroup. For PD and MS, TR seems to yield comparable results to in-person treatment. Further research, adhering as much as possible to the recommendations for correct reporting is essential to explore the impact of TR on QoL in patients with neurological impairments, an aspect not consistently explored in the studies. It is desirable to conduct more in-depth exploration in neurodegenerative pathologies, as TR can serve as a valuable support for chronic conditions and their monitoring.

## Figures and Tables

**Table 1:** Risk of bias in non-RCTs.

Study ID	Confounding Bias	Selection Bias	Classification of Intervention Bias	Deviations from Intended Intervention Bias	Attrition Bias	Detection Bias	Reporting Bias	Overall Bias
Benvenuti et al., 2014 [25]	**Low risk** No confounding domains identified	**Serious risk** Groups mixed and results were combined	**Moderate risk** Participants in the control group were offered to participate to the experimental intervention at the end of their assigned treatment	**Critical risk** 26.5% of dropout; deviations from intended intervention unbalanced between groups	**Critical risk** Missing outcome data; no ITT analysis performed	**Low risk** assessor was not blinded, but the outcome measure was a self-reported questionnaire	**Serious risk** Study protocol not found	**Critical risk** lots of dropout; no ITT; no information on study protocol.
Isernia et al., 2020 [50]	**Low risk** No confounding domains identified	**Low risk** All eligible participants included in the study and followed from the start of the intervention	**Low risk** Intervention status is well defined	**Serious risk** Difference in groups size (ClinicHEAD n.31; UC n. 20; HomeHEAD n. 11). Analysis for baseline differences performed only between UC and HomeHEAD groups	**Low risk** Multiple imputation by chained equations was performed to replace missing values to address potential biases due to incomplete follow-up	**Low risk** self-reported survey	**Low risk** no selection of the reported result found	**Serious risk** some concerns about deviations from intended interventions

## Data Availability

Metadata of this review are available upon request to the corresponding author.

## Supplementary Materials

The following supporting information can be downloaded at: https://www.mdpi.com/article/10.3390/jcm13010299/s1, Table S1. PRISMA 2020 Checklist.

## Appendix A. Search Strategy

**Stroke**

**PUBMED**

#1 (“Stroke”[Mesh] OR “Brain Ischemia”[Mesh] OR “Hemorrhagic Stroke”[Mesh] OR “stroke”[All Fields] OR “cva”[All Fields] OR “post stroke”[All Fields] OR hemiplegia[MeSH] OR cerebrovascular disorders [MeSH] OR basal ganglia cerebrovascular disease [MeSH] OR carotid artery diseases [MeSH] OR intracranial arterial diseases [MeSH] OR intracranial hemorrhages [MeSH] OR “brain injuries” OR “brain injury, chronic” OR poststroke OR “post-stroke”)
#2 ((“Telerehabilitation”[Mesh]) OR (“Telemedicine”[Mesh]) OR (“Telecommunications”[Mesh]) OR (“telehealth”[All Fields]) OR (“telemedicine”[All Fields]) OR (“telerehabilitation”[All Fields]) OR (videoconferenc*) OR (teletreatment*) OR (“teletherapy”[All Fields]) OR “Distance Education” OR Telepractice OR “Virtual Conferenc*” OR “Tele-rehabilitation” OR “Remote Rehabilitation”)
#3 (“randomized controlled trial*” [MeSH Terms] OR “randomized controlled trial*” [tiab] OR “controlled clinical trial*”[tiab] OR “randomized controlled trial*”[ptyp] OR “controlled clinical trial*”[ptyp] OR “quasi-randomized control trial*”)
#1 AND #2 AND #3

**EMBASE**

#1 ((“Stroke”/de) OR (“Brain Ischemia”/de) OR (“Hemorrhagic Stroke”/de) OR (“stroke”) OR (“cva”) OR (“post stroke”) OR (hemiplegia/de) OR cerebrovascular disorders/de OR basal ganglia cerebrovascular disease/de OR carotid artery diseases/de OR intracranial arterial diseases/de OR intracranial hemorrhages/de OR “brain injuries” OR “brain injury, chronic” OR poststroke OR post-stroke)
#2 (“Telerehabilitation”/de OR “Telemedicine”/de OR “Telecommunications”/de OR “telehealth” OR “telemedicine” OR “telerehabilitation” OR videoconferenc* OR teletreatment* OR “teletherapy” OR “Distance Education” OR Telepractice OR “Virtual Conferenc*” OR “Tele-rehabilitation” OR “Remote Rehabilitation”)
#3 (“randomized controlled trial*”/de OR “randomized controlled trial*” OR “controlled clinical trial*” OR “quasi-randomized control trial*”)
#1 AND #2 AND #3

**COCHRANE**

#1 MeSH descriptor: [Stroke] explode all trees
#2 MeSH descriptor: [Brain Ischemia] explode all trees
#3 MeSH descriptor: [Hemorrhagic Stroke] explode all trees
#4 MeSH descriptor: [Hemiplegia] explode all trees
#5 MeSH descriptor: [Cerebrovascular Disorders] explode all trees
#6 MeSH descriptor: [Basal Ganglia Cerebrovascular Disease] explode all trees
#7 MeSH descriptor: [Carotid Artery Diseases] explode all trees
#8 MeSH descriptor: [Intracranial Arterial Diseases] explode all trees
#9 MeSH descriptor: [Intracranial Hemorrhages] explode all trees
#10 (“stroke” OR “cva” OR “post stroke” OR “brain injuries” OR “brain injury, chronic” OR “poststroke” OR “post-stroke”):ti,ab,kw (Word variations have been searched)
#11 MeSH descriptor: [Telerehabilitation] explode all trees
#12 MeSH descriptor: [Telemedicine] explode all trees
#13 MeSH descriptor: [Telecommunications] explode all trees
#14 (“telehealth” OR “telemedicine” OR “telerehabilitation” OR “videoconferenc*” OR “teletreatment*” OR “Teletheraphy” OR “Distance Education” OR “Telepractice” OR “Virtual conferenc*” OR “Tele-rehabilitation” OR “Remote rehabilitation”):ti,ab,kw (Word variations have been searched)
#15 MeSH descriptor: [Randomized Controlled Trial] explode all trees
#16 ((“randomized controlled trial*” OR “controlled clinical trial*” OR “quasi-randomized controlled trial*”)):ti,ab,kw (Word variations have been searched)
#17 #1 OR #2 OR #3 OR #4 OR #5 OR #6 OR #7 OR #8 OR #9 OR #10
#18 #11 OR #12 OR #13 #14
#19 #15 OR #16 602107
#20 #17 AND #18 AND #19

**WEB OF SCIENCE**

TS = (“Stroke” OR “Brain Ischemia” OR “Hemorrhagic Stroke” OR “cva” OR “post stroke” OR hemiplegia OR “cerebrovascular disorders” OR “basal ganglia cerebrovascular disease” OR “carotid artery diseases” OR “intracranial arterial diseases” OR “intracranial hemorrhages” OR “brain injuries” OR “brain injury, chronic” OR poststroke OR ”post-stroke”)
TS = (“Telerehabilitation” OR “Telemedicine” OR “Telecommunications” OR “telehealth” OR videoconferenc* OR teletreatment* OR “teletherapy” OR “Distance Education” OR Telepractice OR “Virtual Conferenc*” OR “Tele-rehabilitation” OR “Remote Rehabilitation”)
WC = (rehabilitation)
TS = (“randomized controlled trial*” OR “controlled clinical trial*” OR “quasi-randomized control trial*”)

**Parkinson Disease**

**PUBMED**

#1 (“Parkinson Disease” [Mesh] OR “Parkinson Disease” OR “Parkinson”)
#2 ((“Telerehabilitation”[Mesh]) OR (“Telemedicine”[Mesh]) OR (“Telecommunications”[Mesh]) OR (“telehealth”[All Fields]) OR (“telemedicine”[All Fields]) OR (“telerehabilitation”[All Fields]) OR (videoconferenc*) OR (teletreatment*) OR (“teletherapy”[All Fields]) OR “Distance Education” OR Telepractice OR “Virtual Conferenc*” OR “Tele-rehabilitation” OR “Remote Rehabilitation”)
#3 “randomized controlled trial*” [MeSH Terms] OR “randomized controlled trial*” [tiab] OR “controlled clinical trial*”[tiab] OR “randomized controlled trial*”[ptyp] OR “controlled clinical trial*”[ptyp] OR “quasi-randomized control trial*”
#1 AND #2 AND #3

**EMBASE**

#1 (“Parkinson Disease”/de OR “Parkinson Disease” OR “Parkinson”)
#2 ((“Telerehabilitation”/de) OR (“Telemedicine”/de) OR (“Telecommunications”/de) OR (“telehealth”) OR (“telemedicine”) OR (“telerehabilitation”) OR (videoconferenc*) OR (teletreatment*) OR (“teletherapy”) OR “Distance Education” OR Telepractice OR “Virtual Conferenc*” OR “Tele-rehabilitation” OR “Remote Rehabilitation”)
#3 “randomized controlled trial*”/de OR “randomized controlled trial*” OR “controlled clinical trial*” OR “quasi-randomized control trial*”
#1 AND #2 AND #3

**WEB OF SCIENCE**

#1 TS = (”Parkinson Disease” OR Parkinson)
#2 TS = (“Telerehabilitation” OR “Telemedicine” OR “Telecommunications” OR “telehealth” OR videoconferenc* OR teletreatment* OR “teletherapy” OR “Distance Education” OR Telepractice OR “Virtual Conferenc*” OR “Tele-rehabilitation” OR “Remote Rehabilitation”)
**#**3 WC = (rehabilitation)
**#**4 TS = (“randomized controlled trial*” OR “controlled clinical trial*” OR “quasi-randomized control trial*”)
#1 AND #2 AND #3 AND #4

**COCHRANE**

#1 MeSH descriptor: [Parkinson Disease] explode all trees
#2 “Parkinson Disease” OR “Parkinson”
#3 MeSH descriptor: [Telerehabilitation] explode all trees
#4 MeSH descriptor: [Telemedicine] explode all trees
#5 MeSH descriptor: [Telecommunications] explode all trees
#6 (“telehealth” OR “telemedicine” OR “telerehabilitation” OR “videoconferenc*” OR “teletreatment*” OR “Teletheraphy” OR “Distance Education” OR “Telepractice” OR “Virtual conferenc*” OR “Tele-rehabilitation” OR “Remote rehabilitation”):ti,ab,kw (Word variations have been searched)
#7 MeSH descriptor: [Randomized Controlled Trial] explode all trees
#8 ((“randomized controlled trial*” OR “controlled clinical trial*” OR “quasi-randomized controlled trial*”)):ti,ab,kw (Word variations have been searched)
#9 #1 OR #2
#10 #3 OR #4 OR #5 OR #6
#11 #7 OR #8
#12 #9 AND #10 AND #11

**Multiple Sclerosis**

**PUBMED**

#1 (“Multiple Sclerosis”[Mesh] OR “Multiple Sclerosis, Chronic Progressive”[Mesh] OR “Multiple Sclerosis” OR “Multiple Sclerosis, Chronic Progressive” OR “Multiple Sclerosis, Relapsing-Remitting”[Mesh] OR “Multiple Sclerosis, Relapsing-Remitting”)
#2 ((“Telerehabilitation”[Mesh]) OR (“Telemedicine”[Mesh]) OR (“Telecommunications”[Mesh]) OR (“telehealth”[All Fields]) OR (“telemedicine”[All Fields]) OR (“telerehabilitation”[All Fields]) OR (videoconferenc*) OR (teletreatment*) OR (“teletherapy”[All Fields]) OR “Distance Education” OR Telepractice OR “Virtual Conferenc*” OR “Tele-rehabilitation” OR “Remote Rehabilitation”)
#3 (“randomized controlled trial*” [MeSH Terms] OR “randomized controlled trial*” [tiab] OR “controlled clinical trial*”[tiab] OR “randomized controlled trial*”[ptyp] OR “controlled clinical trial*”[ptyp] OR “quasi-randomized control trial*”)
#1 AND #2 AND #3

**EMBASE**

#1 (“Multiple Sclerosis”/de OR “Multiple Sclerosis, Chronic Progressive”/de OR “Multiple Sclerosis” OR “Multiple Sclerosis, Chronic Progressive” OR “Multiple Sclerosis, Relapsing-Remitting”/de OR “Multiple Sclerosis, Relapsing-Remitting”)
#2 (“Telerehabilitation”/de OR “Telemedicine”/de OR “Telecommunications”/de OR “telehealth” OR “telemedicine” OR “telerehabilitation” OR videoconferenc* OR teletreatment* OR “teletherapy” OR “Distance Education” OR Telepractice OR “Virtual Conferenc*” OR “Tele-rehabilitation” OR “Remote Rehabilitation”)
#3 (“randomized controlled trial*”/de OR “randomized controlled trial*” OR “controlled clinical trial*” OR “randomized controlled trial*” OR “controlled clinical trial*” OR “quasi-randomized control trial*”)
#1 AND #2 AND #3

**COCHRANE**

#1 MeSH descriptor: [Multiple Sclerosis] explode all trees
#2 MeSH descriptor: [Multiple Sclerosis, Chronic Progressive] explode all trees
#3 MeSH descriptor: [Multiple Sclerosis, Relapsing-Remitting] explode all trees
#4 (“multiple sclerosis” OR “mutiple sclerosis, relapsing-remitting” OR “Multiple sclerosis, Chronic Progressive”):ti,ab,kw
#5 MeSH descriptor: [Telerehabilitation] explode all trees
#6 MeSH descriptor: [Telemedicine] explode all trees
#7 MeSH descriptor: [Telecommunications] explode all trees
#8 (“telehealth” OR “telemedicine” OR “telerehabilitation” OR “videoconferenc*” OR “teletreatment*” OR “Teletheraphy” OR “Distance Education” OR “Telepractice” OR “Virtual conferenc*” OR “Tele-rehabilitation” OR “Remote rehabilitation”):ti,ab,kw (Word variations have been searched)
#9 MeSH descriptor: [Randomized Controlled Trial] explode all trees
#10 ((“randomized controlled trial*” OR “controlled clinical trial*” OR “quasi-randomized controlled trial*”)):ti,ab,kw (Word variations have been searched)
#11 #1 OR #2 OR #3 OR #4
#12 #5 OR #6 OR #7 OR #8
#13 #9 OR #10
#14 #11 AND #12 AND #13

**WEB OF SCIENCE**

#1 TS = (”Multiple Sclerosis” OR “Multiple Sclerosis, Chronic Progressive” OR “Multiple Sclerosis, Relapsing-Remitting”)
#2 TS = (“Telerehabilitation” OR “Telemedicine” OR “Telecommunications” OR “telehealth” OR videoconferenc* OR teletreatment* OR “teletherapy” OR “Distance Education” OR Telepractice OR “Virtual Conferenc*” OR “Tele-rehabilitation” OR “Remote Rehabilitation”)
#3 WC = (rehabilitation)
#4 TS = (“randomized controlled trial*” OR “controlled clinical trial*” OR “quasi-randomized control trial*”)
#1 AND #2 AND #3 AND #4

## Appendix B

**Table A1:** Synthesis of satisfaction and acceptance outcomes across the studies.

Pathology	Study	Satisfaction	Outcome Measures	Findings	Acceptance	Outcome Measures	Findings
**Stroke**	Asano et al., 2018 [24]	N/A *	N/A	N/A	Present	Expressed as a calculation of hours of therapy sessions conducted.	There was no significant difference in the median time spent on rehabilitation and exercise between the two groups.
	Benvenuti et al., 2014 [25]	Present	Structured interviews using Likert-type scales were administered to participants and caregivers.	The intervention received high satisfaction ratings and produced no adverse events.	Present	An evaluation questionnaire augmented by several dimensions from the Unified Theory of User Acceptance and Use of Technology (UTAUT).	50 subjects were highly adherent to the study protocol, 88 demonstrated average adherence, and 30 low adherence.
	Cadilhac et al., 2020 [26]	Present	Survey utilizing closed and open question formats. Participants provided feedback on program aspects (benefits, willingness to continue, and likelihood of recommending to other stroke survivors). The intervention group shared input on electronic health support (message details, support duration).	More than 85% of participants in both groups found the goal-setting form beneficial for developing their goals. Additionally, both the intervention (92%) and control (72%) groups agreed that clinicians were helpful in goal development, with a non-significant difference.	Present	Survey utilizing closed and open question formats. Perceived benefit of the electronic health support (intervention group only).	No unintended harms or effects were reported. In total, 77% of participants believed that text or email messages helped them to achieve their goals and were a good way to receive education about stroke. Participants were comfortable with technology use and felt that the system was easy to understand.
	Chumbler et al., 2015 [29]	Present	Stroke-specific satisfaction With care (SSPSC) questionnaire and interview consisting of 13 closed-ended questions (using a 5-point Likert-type scale) and 4 open-ended questions.	Participants reported a greater effect on hospital satisfaction than home satisfaction. Subjects were satisfied with the in-home intervention, finding it convenient, useful and expressing comfort with being videotaped during sessions.	N/A	N/A	N/A
	Grau-Pellicer et al., 2020 [30]	Present	Ad hoc questionnaire to assess satisfaction in relationship with the benefits obtained (use of app, improvement of physical condition, gait capacity, balance, expectations, and self-efficacy).	Most patients reported a high level of satisfaction, with all expressing a favourable opinion in recommending the treatment to others. No adverse effect was reported.	N/A	N/A	N/A
	Jarbandhan et al., 2022 [31]	Present	A self-developed questionnaire consisting of 7 Likert scale questions and an optional open-ended question for system improvement recommendations.	Participants, in general, had a positive experience with the program. Some of them expressed a preference for a longer duration of the program or sessions.	Present	Expressed through adherence to the treatment.	Participants perceived the intervention as supportive. Adherence to the treatment was influenced by factors such as the rainy season and associated infrastructural issues (n = 2), participants’ medical status (n = 3), and insufficient motivation to continue the program without direct supervision (n = 1). Notably, no adverse events were reported.
	Lin et al., 2014 [32]	Present	A questionnaire survey derived from the “Successes of the technology acceptance model” and the “Model of information systems technology” (David [1989], DeLone and McLean [2003]). Each item was assessed using a Likert scale, and the values for each dimension were obtained by averaging the scores of the corresponding items within that dimension.	Overall, participants in both groups expressed a high level of satisfaction, perception of ease of use, and a positive attitude toward TR system, with a willingness to recommend it to others. The results showed no significant differences in all items, except for perceived usefulness and perceived satisfaction of system in the TR group.	N/A	N/A	N/A
	Piron et al., 2008 [34]	Present	12-item questionnaire, derived from a validated scale (Monnin 2002), measuring the patient’s satisfaction with physical therapy, patient’s attitude towards the treatment, the patient–therapist relationship, and global opinion about the treatment performed. Each item was measured with a Likert scale.	The two groups reported similar levels of satisfaction, with no significant differences in most aspects, including treatment comprehension, equipment, user-friendliness, and the patient–therapist relationship.	N/A	N/A	N/A
	Uswatte et al., 2021 [36]	Present	Participant opinion survey [64] assessing perceptions about the therapeutic value and difficulty of the interventions before and after treatment. This consisted of a 7-point Likert scale and 1 open-ended question.	Participants expressed high satisfaction with the intervention received and moderate satisfaction regarding the perceived difficulty of the intervention.	N/A	N/A	N/A
	Cho et al., 2022 [27]	Present	A 10-item, 7-point Likert scale related to the patients’ satisfaction with the therapy program and the exercises assigned to them. The final session was employed as the indicator of overall satisfaction with the therapy.	The increased use of goal adjustment strategies led to greater satisfaction at the end of therapy, supporting the notion that motivational benefits derived from goal adjustment positively influenced overall satisfaction. Patient satisfaction was not linked to the actual level of arm motor status recovery.	N/A	N/A	N/A
**Parkinson’s Disease**	Cubo et al., 2017 [47]	Present	A visual analogic scale. Collection of the number of technical problems associated with the use of the device.	Treatment was feasible and patients were satisfied when it is was used in conjunction with their regular clinical visits and telephone/email support.	N/A	N/A	N/A
	Flynn et al., 2021 [48]	N/A	N/A	N/A	Present	Examined using a participant questionnaire about the program. Participants were also interviewed about their experiences of exercise at home and in a center.	Questionnaire was completed by 88% of participants at the end of Week 5 and 85% at the end of Week 10. At Week 5, all participants reported finding the exercise helpful, group exercising satisfying, and would recommend it to others. By Week 10, center-based group participants echoed these sentiments. However, home-based group participants found the exercise helpful and could follow the program, but only 53% found it satisfying, and 6% did not recommend it.
	Gandolfi et al., 2017 [49]	Present	A questionnaire investigating domains considered relevant for the patient; responses for each domain were marked on a 5-point Likert-type scale. Patients were provided with a logbook to record their feelings and any difficulties or adverse events they had experienced at each training session.	No statistically significant difference in satisfaction rates between the two groups.	N/A	N/A	N/A
**Multiple Sclerosis**	Dlugonski et al., 2012 [40]	Present	Ad hoc process evaluation questionnaire, containing 5 Likert items and 1 open-ended final feedback.	Participants expressed high satisfaction with the overall program, staff, and the provided pedometer. The satisfaction with the website itself was slightly lower. In open-ended feedback, participants expressed a desire for more interaction with others, but some found the forum section of the website challenging to use. All participants indicated they would recommend the program to others.	N/A	N/A	N/A
	Kratz et al., 2020 [42]	N/A	N/A	N/A	Present	The client satisfaction questionnaire (CSQ-8)	For the experimental group, the treatment was highly feasible and acceptable. Attendance rates were higher for the experimental group.
	Paul et al., 2014 [43]	Present	Ad hoc questionnaire based on Finkelstein 2008.	Treatment was a feasible method for delivering physiotherapy and was deemed acceptable by individuals moderately affected by MS. Evaluation questionnaire responses indicated that the system was easy to use and received high ratings from participants.	Present	Telephone interviews recorded, transcribed, and verified. Emerging themes and sub-themes were identified and agreed between two independent researchers.	Treatment was considered acceptable, usable, and convenient.
	Paul et al., 2019 [44]	N/A	N/A	N/A	Present	Semi-structured telephone interviews with physiotherapists and participants, investigating their reasons for taking part in the study, their views of the assessments and intervention, any issues faced, the perceived benefit, and recommendations for a future trials.	The treatment was feasible and acceptable to both participants and physiotherapists, with no intervention-related adverse events.

Notes: * N/A = indicates that the specific outcome was not utilized or investigated in the respective studies.
